# Supplementary material for: Computational studies reveal structural characterization and novel families of Puccinia striiformis f. sp. tritici effectors
Source: PLoS Comput Biol. 2025 Mar 28;21(3):e1012503. doi: 10.1371/journal.pcbi.1012503 (PMC11952758; doi:10.1371/journal.pcbi.1012503)
Supplement: S1 Fig — The multiple sequence alignment of Tubby-like effector candidates showing in the Fig 3B. The secondary structure features showing above the alignments from the AlphaFold 2 predicted structure of DK0911_02754. The C-terminus of the sequences with blue background indicates the Tubby-like effector candidates from structure cluster No. 30 (Struc.C_30). The C-terminus of the sequences with green background indicates the Tubby-like effector candidates from Struc.C_103. The corresponding position of 68 Cysteine and 189 Cysteine in the sequence of DK0911_02754 indicates the formation of disulfide bond, marking in green ‘1’ below the alignments. (DOCX) [file pcbi.1012503.s001.docx]

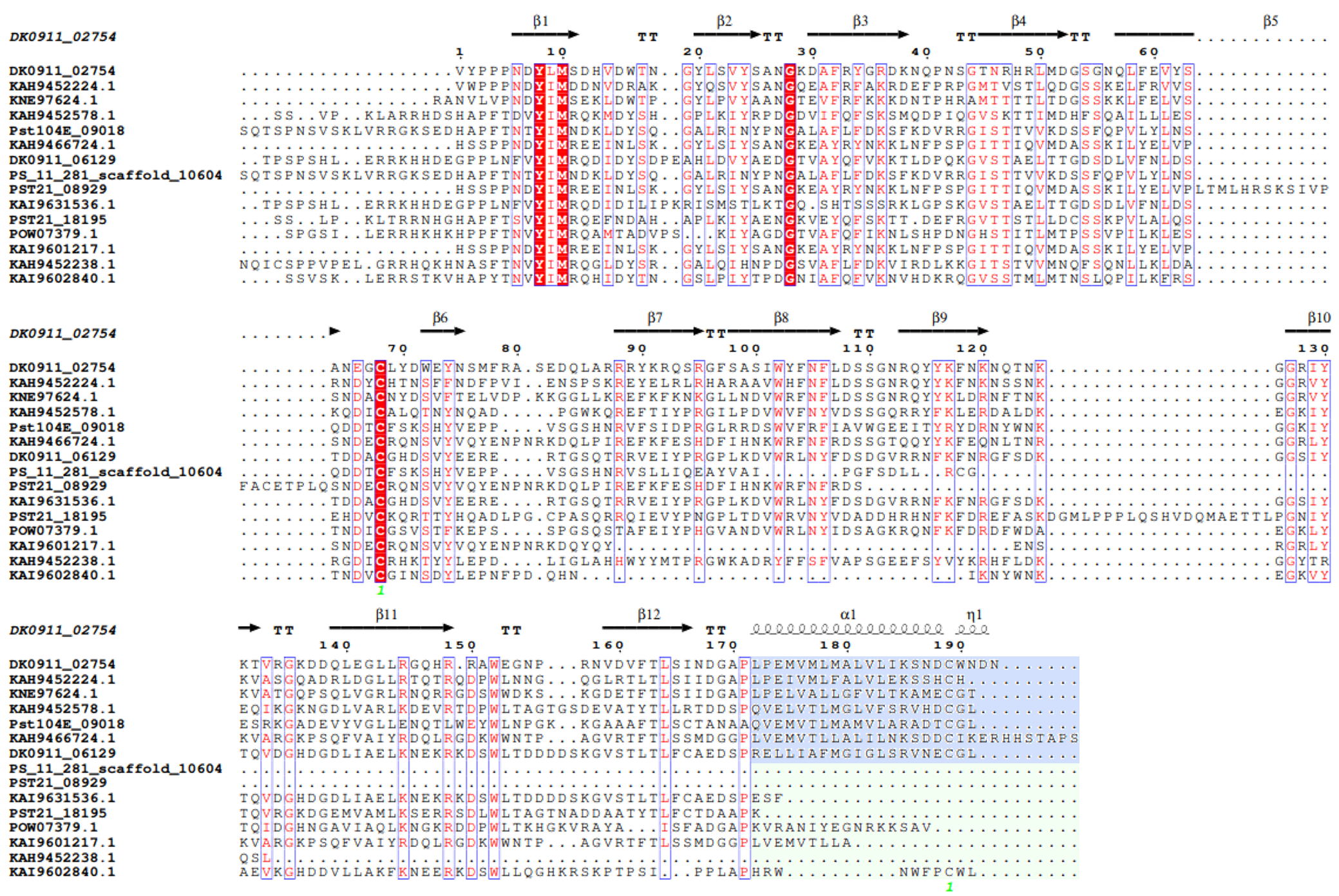


**S1 Fig. The multiple sequence alignment of Tubby-like effector candidates showing in the Fig 3B.** The secondary structure features showing above the alignments from the AlphaFold 2 predicted structure of DK0911_02754. The C-terminus of the sequences with blue background indicates the Tubby-like effector candidates from structure cluster No. 30 (Struc.C_30). The C-terminus of the sequences with green background indicates the Tubby-like effector candidates from Struc.C_103. The corresponding position of 68 Cysteine and 189 Cysteine in the sequence of DK0911_02754 indicates the formation of disulfide bond, marking in green ‘1’ below the alignments.
